# Distinct Processing of Aversive Experience in Amygdala Subregions

**DOI:** 10.1016/j.bpsc.2019.07.008

**Published:** 2020-03

**Authors:** Jochen Michely, Francesco Rigoli, Robb B. Rutledge, Tobias U. Hauser, Raymond J. Dolan

**Affiliations:** a Wellcome Centre for Human Neuroimaging, University College London, London, United Kingdom; b Max Planck UCL Centre for Computational Psychiatry and Ageing Research, University College London, London, United Kingdom; c Department of Psychology, University of London, London, United Kingdom

**Keywords:** Anxiety, Basolateral amygdala, Centromedial amygdala, Emotional processing, fMRI, Threat

## Abstract

**Background:**

The amygdala is an anatomically complex medial temporal brain structure whose subregions are considered to serve distinct functions. However, their precise role in mediating human aversive experience remains ill understood.

**Methods:**

We used functional magnetic resonance imaging in 39 healthy volunteers with varying levels of trait anxiety to assess distinct contributions of the basolateral amygdala (BLA) and centromedial amygdala to anticipation and experience of aversive events. Additionally, we examined the relationship between any identified functional subspecialization and measures of subjective reported aversion and trait anxiety.

**Results:**

Our results show that the centromedial amygdala is responsive to aversive outcomes but insensitive to predictive aversive cues. In contrast, the BLA encodes an aversive prediction error that quantifies whether cues and outcomes are worse than expected. A neural representation within the BLA for distinct threat levels was mirrored in self-reported subjective anxiety across individuals. Furthermore, high trait-anxious individuals were characterized by indiscriminately heightened BLA activity in response to aversive cues, regardless of actual threat level.

**Conclusions:**

Our results demonstrate that amygdala subregions are distinctly engaged in processing of aversive experience, with elevated and undifferentiated BLA responses to threat emerging as a potential neurobiological mediator of vulnerability to anxiety disorders.

Fear and anxiety are adaptive responses to demands of everyday life, such as environmental threat. When these aversive responses are exaggerated, they may lead to a range of anxiety disorders (1). However, it remains unclear why human subjects differ so strikingly in their subjective response to objectively similar threats and in turn in the expression of anxiety traits (2).

The amygdala is a key structure for processing aversive experience and negative emotional information 3, 4. Previous research has highlighted its relevance for threat processing, ascribing to it a role in the genesis of disorders that encompass the anxiety spectrum 5, 6. The amygdala is anatomically heterogeneous, with distinct subregions assumed to serve different functional roles (7). At least 2 major functional subregions can be identified, the basolateral amygdala (BLA) and centromedial amygdala (CMA) 8, 9. Despite substantial evidence derived from nonhuman animal experiments (10) and human anatomical studies 11, 12, little is known regarding a functional subspecialization within human amygdala nuclei (13). Recent neuroimaging studies suggest that the CMA and BLA might have distinct functional roles in humans, primarily in the context of associative learning 14, 15, threat prioritization (16), and social functioning 17, 18.

However, how human amygdala subregions process aversive events, and how expectations about these events modulate these functions, remains unknown. Moreover, owing to the fact that subjective experience cannot be assessed in nonhuman animal models, it remains elusive how amygdala subregions mediate a transformation from objective threat to subjective aversion. Thus, the goal of our study was twofold. First, we combined functional magnetic resonance imaging (fMRI) with a novel Pavlovian conditioning paradigm to probe the exact roles of the BLA and CMA in threat processing, i.e., aversive expectation. Second, we assessed how both interindividual variability and trial-by-trial variability in aversive signals in the amygdala relate to both subjective and trait anxiety. Given its substantial sensory afferent information and implication in threat processing in nonhuman animals, we hypothesized that the BLA, but not the CMA, would encode threat expectations 19, 20. Moreover, we conjectured such a neural signature of threat within the BLA to be related to interindividual differences in anxiety traits (21). Ultimately, owing to its role as the major amygdala output center and in the generation of responses to acute stressors such as pain 22, 23, 24, we assumed CMA activity in response to aversive events to be reflected in subjective reports of aversion.

## Methods and Materials

### Participants

Forty-two healthy, right-handed volunteers (screened for neurological and psychiatric conditions, including anxiety disorders and phobias) participated in this experiment and received monetary compensation for their time (£30–£40). Participants were recruited along usual guidelines from an online subject pool at University College London, but not through courses or lectures given by the authors. Data from 3 subjects were excluded owing to equipment failure involving electrical stimulation during scanning, leaving 39 participants for all subsequent behavioral and neural analyses (mean age 25 years; range, 18–39 years; 22 women). The experimental protocol was approved by the University College London Research Ethics Committee, and informed consent was obtained from all participants.

### Trait Anxiety

To measure trait anxiety, subjects filled out the Spielberger State-Trait Anxiety Inventory trait subscale after the scan, a self-report questionnaire of high internal consistency (Cronbach’s α in present sample: α = .92) that is commonly used to measure anxiety in clinical and nonclinical samples 25, 26. The score ranges between 20 and 80, with higher scores indicating greater trait anxiety.

### Experimental Task

**Figure 1:**
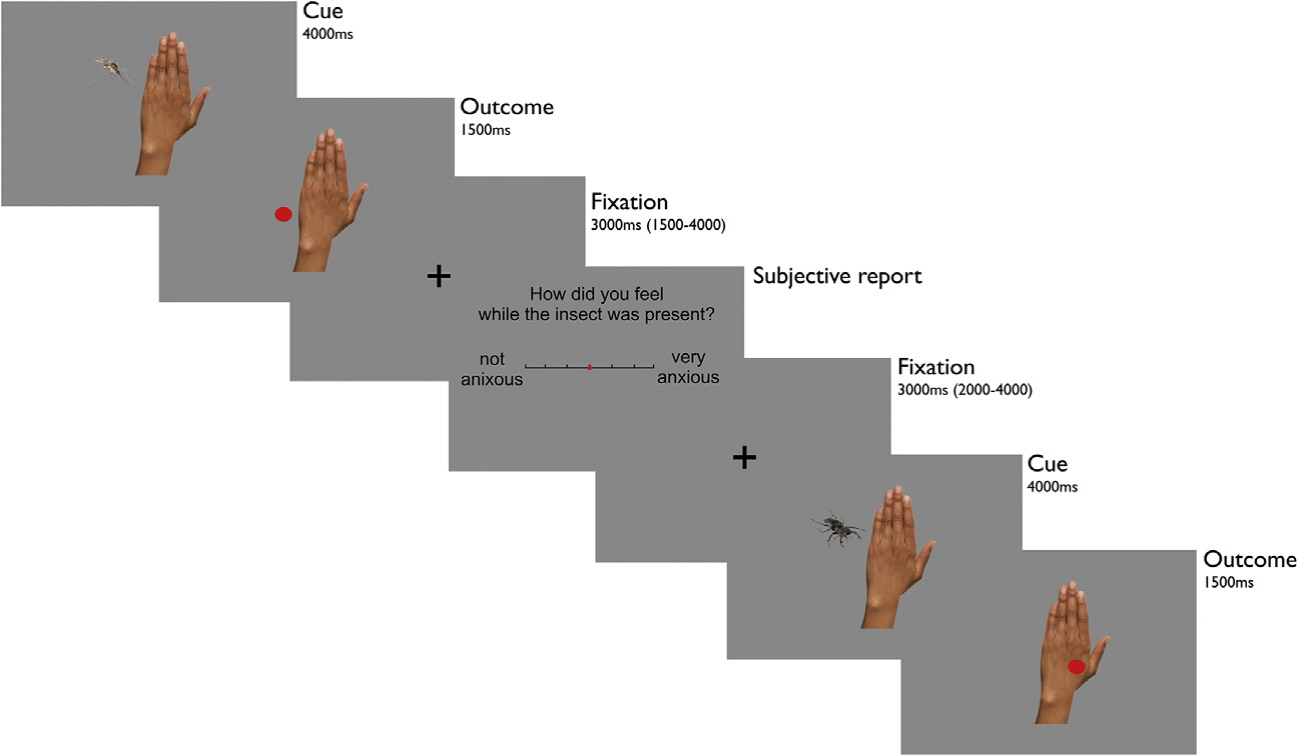
Experimental task. At cue presentation, one of two insects (mosquito or bug) appearing next to a hand indexed an objective probability, learned before the scanning session, of an upcoming electrical shock. One insect indicated a high probability and one insect indicated a low probability of receiving a shock. At outcome, an appearance of a red dot superimposed on the hand indicated receipt of concurrent shock. By contrast, a red dot next to the hand indicated no shock. Following a jittered fixation, subjects were asked to report how anxious they remembered feeling during cue presentations, i.e., while the insect had been present. After another jittered fixation, one of the two insects appeared again to indicate the beginning of the next trial.

We aimed to characterize how participants anticipate and process aversive events (painful electric shocks to the hand) and how neural signals in response to threat relate to subjective experience and trait anxiety. To this end, we designed a novel task consisting of 180 trials, divided into 4 blocks of 45 trials each. On each trial, subjects were presented with a picture of an insect, either a mosquito or a bug, shown next to the back of an image of a hand for 4000 ms (Figure 1). Each insect signaled a specific probability of receiving an electrical shock, with one insect (high-probability cue, 90 trials) followed by a shock on 70% of trials (63 shocks) and by no shock on 30% of trials, and the other insect (low-probability cue, 90 trials) followed by a shock on 30% of trials (27 shocks) and no shock on 70% of trials (insects were counterbalanced across subjects). Note that cues were perfectly matched with respect to their uncertainty (absolute deviation from probability of shock equal to 50%), differing solely in objective predictiveness of shock receipt.

To avoid any influence arising out of learning, subjects were familiarized with shock probability attributed to each stimulus in a prescanning training session and explicitly informed that probabilities remained fixed throughout the entirety of training and experiment. Outcome onset was indicated by the appearance of a red dot (duration 1500 ms), either displayed next to the hand (indicating no shock) or superimposed on the hand (indicating shock; shock duration 100 ms). If the red dot was displayed on the hand, shocks were applied simultaneous in time to its appearance.

After a jittered fixation cross (mean 3000 ms; uniformly distributed between 1500 and 4500 ms), subjects were asked to rate their anticipatory anxiety using a slider (“How anxious did you feel while the insect was present?”). Importantly, subjects were instructed and previously trained to recall the subjective state and feelings elicited by predictive cue presentation, ignoring the actual outcome of each trial. Ratings were given without time restrictions by moving a cursor (always starting at the midpoint) along a scale that spanned “not anxious” on the extreme left to “very anxious” on the extreme right. After another jittered fixation (mean 3000 ms; range, 2000–4000 ms), the appearance of the hand and insect indicated the beginning of the next trial.

On average, 1 block lasted 11 minutes, with minor variation between subjects depending on times for self-paced anxiety ratings. Between blocks, subjects were allowed a break, and we repeated a short pain titration procedure (see below for details). Overall, the task in the scanner lasted on average 55 minutes.

### Electrical Stimulation

Participants underwent an individually tailored pain titration procedure 27, 28 with a Digitimer DS7A electric stimulator (Digitimer Ltd., Welwyn Garden City, UK) that can produce stimulator output as high as 100 mA. An electrode was placed on the back of the subject’s left hand, and titration began with a low-current electric shock (0.1 mA), where subjects were asked to rate its painfulness on a visual 21-point scale (ranging from 0 = not unpleasant to 5 = quite unpleasant to 10 = extremely unpleasant and unbearable). For each subsequent shock, intensity was increased in small increments with the subject’s approval. This procedure was repeated until subjective ratings of pain reached 7 (very unpleasant but bearable). This intensity was used for the first block of the experiment. To avoid excessive habituation to stimulation and excessive pain owing to increased shock sensitivity over the course of the experiment, a short titration procedure was repeated within the scanner before each of the 4 experimental blocks. Hence, perceived subjective experience was kept constant throughout the experiment. Mean shock intensity across subjects was 2.3 ± 1.3 mA (range, 0.5–6.7 mA), and there was no relationship between chosen intensity and trait anxiety (*r* = −.094, *p* = .569).

### Prescanning Training

Subjects performed 2 practice blocks (20 trials each) outside the scanner. This ensured that they had learned the 2 levels of shock probability and familiarized themselves with the task structure. Importantly, subjects were informed beforehand that one of the insects would be associated with a high chance of predicting an upcoming shock and the other would be associated with a low chance of predicting an upcoming shock.

Whereas the first block familiarized subjects with stimuli and associated shock probabilities, i.e., without provision of subjective ratings, the second block was the same as in the scanner, including subjective ratings. Analysis of the second training block indicated that subjects had learned to dissociate the two threat stimuli before entering the scanner, as indicated by a strong difference in anxiety ratings for high- versus low-threat stimuli (Supplemental Figure S1).

### fMRI Data Acquisition and Preprocessing

Data from T2*-weighted echo-planar images (EPIs) were acquired on a Siemens Trio 3T MRI scanner (Siemens Healthcare, Erlangen, Germany) using a 32-channel head coil. We collected whole-brain data, 42 slices with 3-mm isotropic voxels with repetition time = 2.94 seconds; echo time = 30 ms; slice tilt = −30° (transversal > coronal) relative to scanner axis; and z-shim = −0.4. This sequence is designed for optimal sensitivity and reduced susceptibility-induced signal dropout particularly in temporal regions such as the amygdala (29). To account for T1-saturation effects, the first 6 volumes of each session were discarded. Additionally, whole-brain field maps (3-mm isotropic, 10 ms/12.46 ms echo time for short/long, 37 ms total EPI readout time, phase-encode blip polarity −1) were acquired to correct EPIs for field strength inhomogeneity. All fMRI analyses were performed using default settings within SPM12 (Wellcome Centre for Human Neuroimaging; www.fil.ion.ucl.ac.uk). EPIs were realigned and unwarped using the field maps, subsequently coregistered to subject-specific anatomical images, and normalized to Montreal Neurological Institute space using the 1.5-mm MNI152 atlas implemented in SPM12. Finally, normalized EPIs were smoothed with a 6-mm full width at half maximum kernel to satisfy smoothness assumptions of statistical correction algorithms. To ascertain that these results were robust, we conducted additional analyses using reduced smoothing kernels (4.5 mm, 3 mm), which yielded similar results (Supplemental Table S1).

### Structural MRI Data Acquisition

Structural images were acquired using quantitative multiparameter maps in a three-dimensional multiecho fast low-angle-shot sequence with 1-mm isotropic resolution (30). Three different fast low-angle-shot datasets were acquired: predominantly magnetization transfer weighting (repetition time/α = 23.7 ms/6°; excitation preceded by an off-resonance Gaussian magnetization transfer pulse of 4-ms duration, 220° nominal flip angle, and 2-kHz frequency offset), proton density weighting (23.7 ms/6°), and T1 weighting (18.7 ms/20°). To increase signal-to-noise ratio, signals of 6 equidistant bipolar gradient echoes (echo time 2.2–14.7 ms) were averaged. Semiquantitative magnetization transfer maps were calculated using mean signal amplitude and T1 maps (31), additionally eliminating influence of B1 inhomogeneity and relaxation effects (32).

### Behavioral Analysis

To assess what influenced subjects’ anxiety ratings, we ran an analysis of variance (ANOVA) with factors expectation (high/low) and outcome (shock/no shock). Additionally, we fitted a trial-by-trial linear regression model. To predict anxiety ratings on current trial T, we used 1) probability (high vs. low), 2) outcome type (shock vs. no shock), 3) interaction term, 4) elapsed time between outcome offset and rating onset, and 5) rating time (from ratings onset to offset), while also testing for influence of (items 4 and 5) outcome type of previous trials, i.e., trial T−1 and T−2. Behavioral analyses were conducted in MATLAB version 2014a (The MathWorks, Inc., Natick, MA) and IBM SPSS version 25 (IBM Corp., Armonk, NY).

### fMRI Analysis

The main goal of our fMRI analysis was to characterize how different amygdala subregions respond to aversive states, i.e., anticipation and experience of negative outcomes. In a general linear model, we entered 2 different regressors at time of cue, for high and low probability of upcoming shock, respectively. At time of outcome, we entered 4 regressors, separating high-expectation shock, high-expectation no shock, low-expectation shock, and low-expectation no shock. Additionally, we entered 4 equivalent regressors at time of subjective rating period onset. To examine how amygdala responses at the actual time of rating relate to subjective anxiety ratings on a trial-by-trial basis, we used parametric modulators containing trial-by-trial ratings for each regressor, i.e., each condition separately. Hence, we assessed the relationship between amygdala activity and subjective reports regardless of the condition that subjects were in.

Note that first-level regressors were modeled as events, i.e., 0-second duration, and convolved with SPM’s canonical hemodynamic response function as in previous studies assessing amygdala activity in event-related designs [e.g., 15, 26, 33]. We regressed out movement-related variance using 6 head motion parameters as assessed by the realignment algorithm. Each run was modeled as a separate session to account for offset differences in signal intensity.

To assess activity in amygdala subregions, we used cytoarchitectonically demarcated probabilistic maps, focusing specifically on centromedial (CMA; central and medial nuclei) and basolateral (BLA; lateral, basolateral, basomedial, and paralaminar nuclei) nuclear groups (34). Masks were created via the SPM anatomy toolbox, i.e., cytoarchitectonically defined by using maximum probability maps, representing summary maps of different probabilistic cytoarchitectonic maps 34, 35. One of the advantages of these maps is that they allow the definition of a continuous volume for a subregion without any overlap with other subregions. Another advantage is that the procedure accords with existing fMRI studies on human amygdala subspecialization using similar methods [e.g., 15, 33, 36, 37]. We refer to this parcellation as CMA and BLA masks in Results (see Supplemental Figure S2 for detailed visualization of the masks).

To demonstrate the robustness of task-activated amygdala responses without the restrictions of a parcellation approach, we also used a bilateral anatomical mask for the entire amygdala from the WFU PickAtlas toolbox in SPM, defined by using the automated anatomical labeling atlas. We refer to this independent mask as the entire amygdala in Results. To correct for multiple comparisons, we used a familywise error (FWE) rate threshold of *p* < .05, small volume corrected for predefined bilateral regions of interest (uncorrected height threshold *p* < .001). Figures of whole-brain maps at the respective height threshold are presented in Supplemental Figure S6. Additionally, we report activations surviving at *p* < .05 FWE-corrected for the whole brain. Activations are reported using x, y, z coordinates in Montreal Neurological Institute space.

## Results

### Retrospective Anxiety as a Function of Actual Threat and Experienced Outcomes

To assess how an objective threat is transformed into the subjective experience of anxiety, we asked subjects to report how anxious they felt during predictive cue presentation, i.e., before outcomes were revealed. Importantly, we probed subjects after outcome delivery by specifically asking for a subjective judgment about feelings at cue presentation. As per instruction, these self-reports should not be influenced by actual outcomes. We ran an ANOVA with factors expectation (high/low) and outcome (shock/no shock). This revealed significant main effects of expectation (*F*_1,38_ = 103.505, *p* < .001) and outcome (*F*_1,38_ = 24.430, *p* < .001), with no interaction (*F*_1,38_ = 2.153, *p* = .151). An additional trial-by-trial linear regression model confirmed these results, while additionally showing no effect of outcome history, elapsed time since outcome receipt, or time taken to report (Supplemental Figure S3). Importantly, a separate analysis of the first and second halves of the experiment indicated that anxiety ratings were remarkably stable across halves (Supplemental Figure S1). Findings indicate anxiety ratings were both strongly influenced by objective threat level, i.e., greater for high versus low expectation of upcoming shock, and biased by experienced outcomes, i.e., greater for shock versus no shock trials. Thus, not only did subjects dissociate between objectively different threat levels at cue, but also their subjective reports of anxiety were distorted by a recent receipt of an aversive outcome.

### Threat Dissociation During Aversive Anticipation in BLA

**Figure 2:**
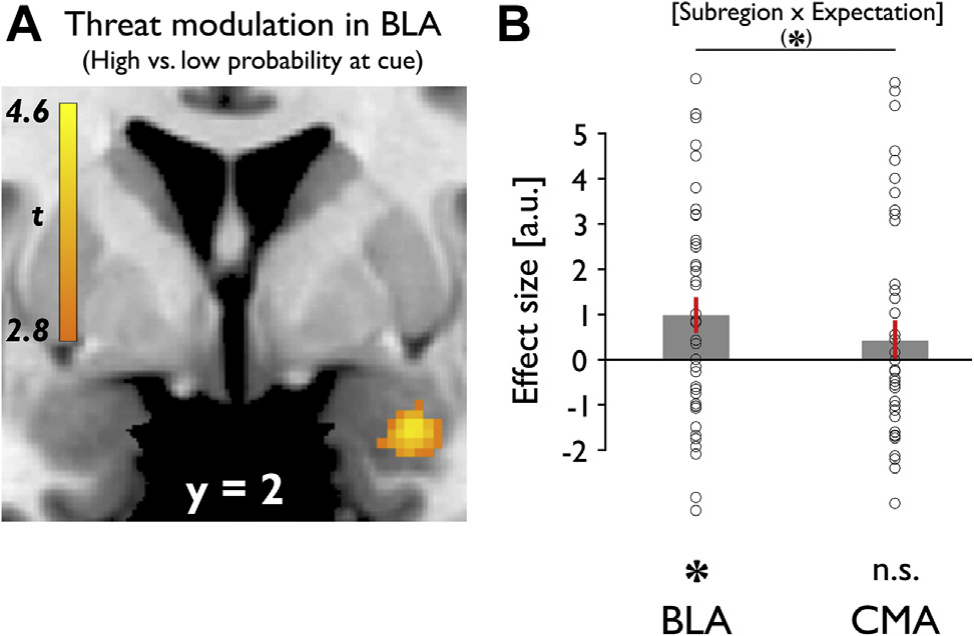
Amygdala responses to different levels of threat at cue presentation. **(A)** Greater basolateral amygdala (BLA) activity at time of cue presentation was associated with enhanced objective threat levels, i.e., high vs. low probability of upcoming shock. **(B)** Trend-level interaction between subregion and expectation at cue, with significant threat modulation (high vs. low probability of upcoming shock) in BLA and no effect in centromedial amygdala (CMA). Mean β values for bilateral BLA and CMA masks. *p < .05, (*)*p* = .074. Error bars indicate SEM. Neural results are presented as SPM activation maps overlaid on a default structural brain in MRIcron (75). a.u., arbitrary units; n.s., not significant.

To investigate how amygdala subregions responded to objective threat, we used a voxel-based analysis, comparing cue-elicited responses signaling high versus low probability of upcoming shocks. The BLA showed a significant dissociation for threat levels, with a significantly enhanced response to high- compared with low-shock-probability cues ([29, 3, −24], *t*_38_ = 4.47, *p*_*FWE*_ = .018 BLA, *p*_*FWE*_ = .028 entire amygdala) (Figure 2A). A control analysis using a finite impulse response set showed a remarkably similar result (Supplemental Figure S4), confirming that the BLA threat response was accurately modeled using a canonical hemodynamic response function. We did not find such threat level modulation in the CMA (even at an uncorrected height threshold of *p* < .001), suggesting a functional dissociation with only the BLA processing threat.

To formally assess a dissociation in responsivity across subregions, we compared mean activation at cue for both subregions. For this analysis, we extracted average β values across all voxels within bilateral anatomical masks. A repeated measures ANOVA, with factors subregion (BLA/CMA) and expectation (high/low), showed no effect of subregion (*F*_1,38_ = 0.094, *p* = .760), a statistical trend for expectation (*F*_1,38_ = 3.209, *p* = .081), and a trend-level interaction (*F*_1,38_ = 3.379, *p* = .074). Follow-up *t* tests showed BLA responses were greater for high as compared with low probability of upcoming shock (*t*_38_ = 2.504, *p* = .017), whereas no such effect was evident in the CMA (*t*_38_ = 0.932, *p* = .357) (Figure 2B). These results suggest the BLA shows a modulation in response across threat levels during anticipation of aversive outcomes.

### Dissociable Response in BLA and CMA to Aversive Outcomes

**Figure 3:**
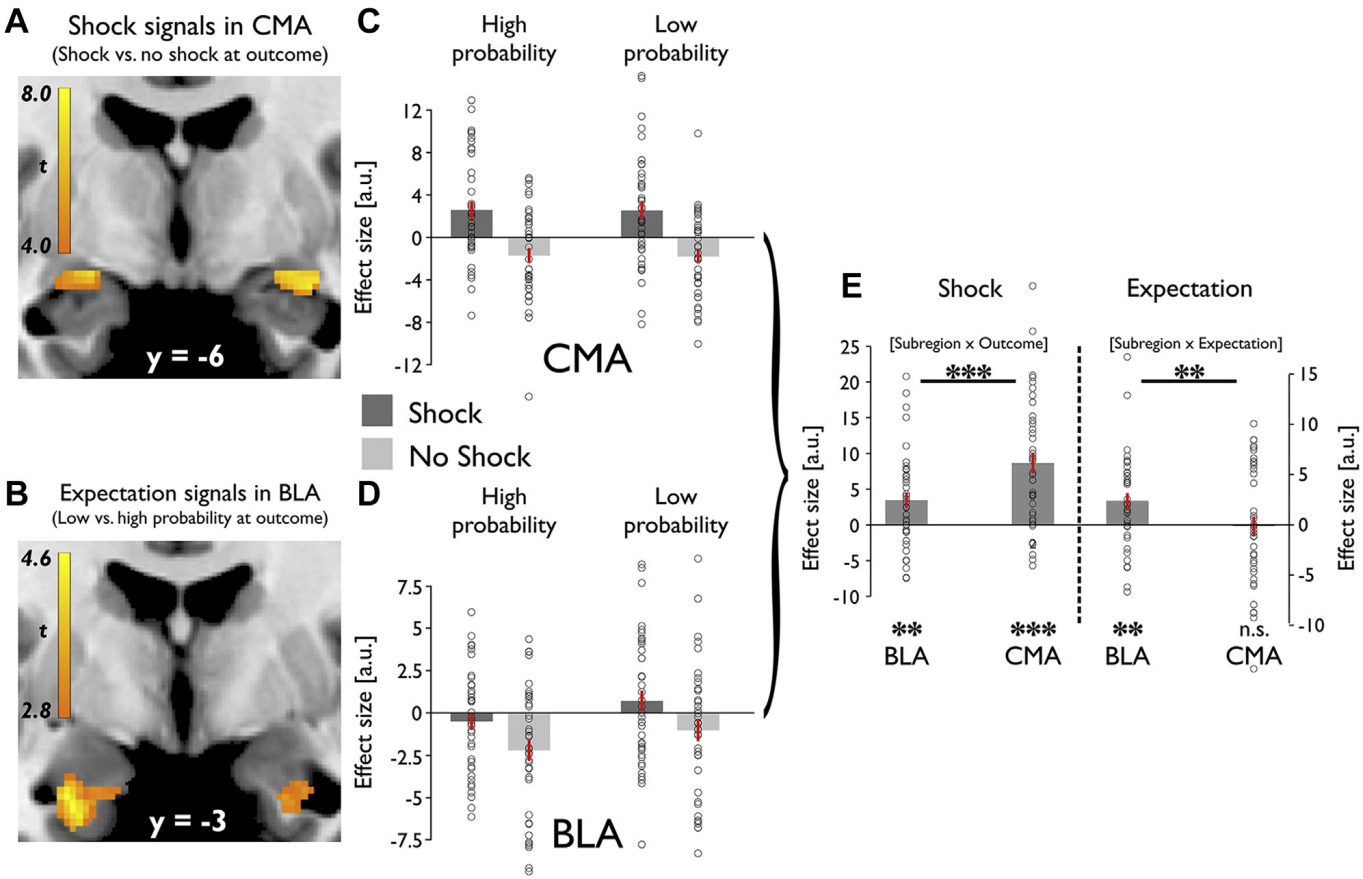
Dissociation between basolateral amygdala (BLA) and centromedial amygdala (CMA) in response to aversive events. **(A)** Shock vs. no shock outcomes are associated with increased outcome-related activity in the CMA. **(B)** Low- vs. high-expectation cues are associated with increased activity in the BLA at the time of outcomes, contrasting with expectation-related modulation at the time of cue presentation. **(C)** Response to all 4 outcome types in CMA. Activity in CMA represents aversive events, which are not modulated by expectations. Mean β values for bilateral CMA mask. High probability and low probability indicate high and low probability of shock. **(D)** Response to all 4 outcome types in BLA. Activity in BLA represents an aversive prediction error that depends on both aversive events and expectations about those events. Stronger activation for less predicted (low probability of shock) compared with highly predicted (high probability of shock) aversive events. Stronger attenuation of responses for less predicted (high probability of shock) compared with highly predicted (low probability of shock) omission of aversive events. Mean β values for bilateral BLA mask. High probability and low probability indicate high and low probability of shock. **(E)** Significant interaction between subregion and outcome as indicated by greater shock responses in CMA than BLA. Significant interaction between subregion and expectation as indicated by significant effect of expectation in BLA and no effect in CMA. Mean β values for bilateral BLA and CMA masks. ***p < .001, **p < .01, *p < .05. Error bars indicate SEM. a.u., arbitrary units, n.s., not significant.

The amygdala represents aversive outcomes, and these responses are thought to be modulated by expectation 15, 38. Thus, we asked how the BLA and CMA respond to aversive events and whether there was a modulation by expectation related to these same events. We first compared activity for shock versus no shock outcomes, regardless of prior expectation. A voxel-based analysis revealed significant activation in the amygdala, with bilateral peaks centered on the CMA ([−20, −6, −12], *t*_38_ = 7.20 and [26, −9, −12], *t*_38_ = 7.07, *p*_*FWE*_ < .001 CMA, *p*_*FWE*_ < .001 entire amygdala) (Figure 3A). Significant shock responses were also found in bilateral BLA ([26, 3, −21], *t*_38_ = 5.83, *p*_*FWE*_ < .001 BLA; [−24, −2, −20], *t*_38_ = 4.79, *p*_*FWE*_ = .007 BLA). Extending this analysis to the whole brain, we found areas encompassing a so-called pain matrix responding more to shocks than no shock conditions (including bilateral insula, adjacent somatosensory cortex, medial/anterior cingulate cortex, periaqueductal gray, thalamus, and amygdala; *p* < .05 whole-brain FWE-corrected) (Supplemental Figure S5; Supplemental Table S3). To assess whether head motion could account for neural shock signals, we assessed framewise displacement during our task. The comparison of framewise displacement for shock versus no shock period showed no difference (1 volume post outcome onset: *p* = .581, 3 volumes: *p* = .206, 5 volumes: *p* = .400). Thus, head movements during shock delivery did not account for these findings.

Next, we assessed whether expectation modulated outcome processing. We found an expectation effect on BLA responses to outcomes ([−26, −2, −30], *t*_38_ = 4.44, *p*_*FWE*_ = .020 BLA, *p*_*FWE*_ = .016 entire amygdala) (Figure 3B), with greater activation for low versus high expectation trials. Such an expectation-induced effect was not evident in the CMA. This suggests that whereas both subregions respond to shock, the BLA alone encodes an expectation of shock outcomes.

To assess this functional dissociation more formally, we examined mean activation for all 4 outcome types within bilateral anatomical masks (Figure 3C, D). A repeated measures ANOVA with factors subregion (BLA/CMA), expectation (high/low), and outcome (shock/no shock) showed an effect of subregion (*F*_1,38_ = 5.614, *p* = .023) and outcome (*F*_1,38_ = 24.652, *p* < .001), but no effect of expectation (*F*_1,38_ = 2.182, *p* = .148). We identified a significant interaction between subregion and expectation (*F*_1,38_ = 10.082, *p* = .003) and between subregion and outcome (*F*_1,38_ = 43.206, *p* < .001). Post hoc *t* tests confirmed that whereas both subregions responded significantly to shock (BLA: *t*_38_ = 3.167, *p* = .003; CMA: *t*_38_ = 5.965, *p* < .001) (Figure 3E), this response was significantly greater in the CMA than the BLA (*t*_38_ = 6.573, *p* < .001). In contrast, the BLA alone encoded expectation (BLA: *t*_38_ = 3.169, *p* = .003; CMA: *t*_38_ = −0.144, *p* = .886) (Figure 3E), indicated by a significant interaction between subregion and expectation, reflecting an effect greater for the BLA than the CMA (*t*_38_ = 3.175, *p* = .003). There was no significant 3-way interaction (*F*_1,38_ = 0.003, *p* = .956), indicative of the 2-way interactions representing 2 separate effects. Overall, the profile of the BLA response fulfilled requirements for a signed aversive prediction error (39), with enhanced response for less compared with highly predicted aversive events (*t*_38_ = 2.343, *p* = .024) and an attenuated response for less compared with a highly predicted aversive event omission (*t*_38_ = 2.232, *p* = .032) (Figure 3D).

### Amygdala Activity and Subjective Aversive Experience

**Figure 4:**
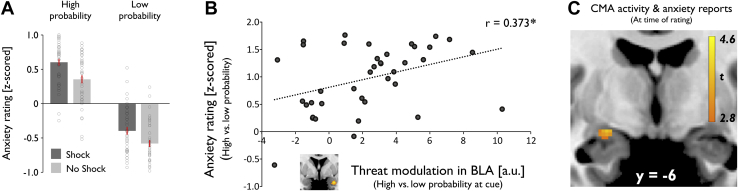
Amygdala activity and subjective aversive experience. **(A)** Subjective reports of remembered anticipatory anxiety at cue presentation. Anxiety ratings both were influenced by objective threat level in the cue period, i.e., greater for high vs. low expectation of upcoming shock, and were biased by experienced outcomes, i.e., greater for shock vs. no shock trials. Error bars indicate SEM. **(B)** A greater neural difference between cue-elicited basolateral amygdala (BLA) responses (high vs. low probability of upcoming shock) was linked to a greater dissociation between threat levels in anxiety ratings (high vs. low probability of shock). *p < .05. **(C)** Positive correlation between trial-by-trial variability in centromedial amygdala (CMA) activity at time of reporting on a visual analog scale and retrospective reports of subjective anxiety at cue presentation. a.u., arbitrary units.

As highlighted above, retrospective reports of cue-elicited anxiety were influenced both by objective threat level at cue and by outcomes (Figure 4A). Consequently, we asked how amygdala activity in response to threat and aversive outcomes related to reports of aversive experience. First, we examined whether the neural dissociation between threat levels in the BLA at cue related to a corresponding effect of expectation on self-reported anxiety. We found that threat-related modulation of BLA activity (high vs. low objective probability of upcoming shock) correlated with an equivalent dissociation of threat levels in subjective reports (high vs. low objective probability of shock, peak voxel activity) (*r* = .373, *p* = .020) (Figure 4B). Notably, this relationship remained significant when controlling for CMA activity (*r* = .357, *p* = .028). However, there was no such relationship for CMA activity alone (*r* = .113, *p* = .491). This indicates that greater threat-related modulation of BLA activity is mirrored in a behavioral dissociation of threat-induced subjective anxiety across participants.

Next, we assessed whether shock effects at outcome as observed in both BLA and CMA related to corresponding distorting outcome effects on retrospective anxiety reports. We found no significant relationship (shock vs. no shock, peak voxel activity: CMA: *r* = .076, *p* = .645; BLA: *r* = .101, *p* = .542), indicating no systematic impact of amygdala shock responses and subjective reports across participants.

Finally, we tested whether amygdala responses at the actual time of rating, i.e., when aversive experience was retrospectively constructed related to how anxious subjects reported to have felt. Importantly, we used a separate parametric modulator for each of the 4 conditions at rating period onset. Thus, we regressed out main effects of cues and outcomes so as to control for the known impact of expectation and shock, assessing the relationship between amygdala activity and subjective reports regardless of the condition that subjects were in. CMA activity positively correlated with subjective anxiety reports ([−23, −6, −12], *t*_38_ = 4.14, *p*_*FWE*_ = .011 CMA, *p*_*FWE*_ = .032 entire amygdala) (Figure 4C). There was no such effect in the BLA. A repeated measures ANOVA with factors subregion (BLA/CMA), expectation (high/low), and outcome (shock/no shock) to confirm a functional subspecialization showed an effect of subregion (*F*_1,38_ = 6.700, *p* = .014) and a significant subregion outcome interaction (*F*_1,38_ = 8.068, *p* = .007). Follow-up *t* tests confirmed a significant effect in the CMA (*t*_38_ = 2.232, *p* = .032), but not the BLA (*t*_38_ = 0.512, *p* = .611) (Supplemental Figure S7). Moreover, the effect was significantly greater in the CMA than the BLA (*t*_38_ = 2.588, *p* = .014), particularly after shock compared with no shock outcomes (*t*_38_ = 2.841, *p* = .007). This suggests postshock CMA activity when making retrospective anxiety reports, i.e., after outcomes were revealed, biased the recollected subjective experience of previous anticipatory aversive states.

### Threat Signals in BLA Relate to Trait Anxiety

Previous research has reported amygdala hyperactivity in highly anxious individuals across a range of experimental paradigms 40, 41, 42. However, the exact relationship between amygdala responses to threat and trait anxiety remains unclear. One hypothesis proposes that anxious individuals do not regulate amygdala responses to variable levels of threat, thus exhibiting indiscriminately heightened amygdala activation 43, 44, 45.

**Figure 5:**
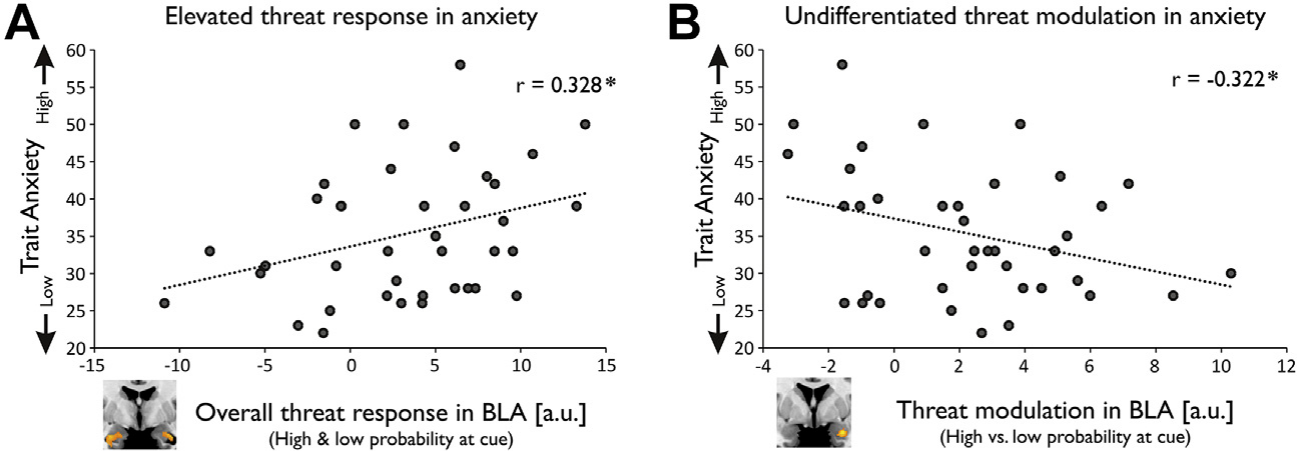
Threat signals in basolateral amygdala (BLA) and trait anxiety. **(A)** A greater overall cue-related BLA response (high and low probability of upcoming shock) was associated with greater trait anxiety. **(B)** A greater neural difference between cue-related BLA responses (high vs. low probability of upcoming shock) was associated with lower trait anxiety. *p < .05. a.u., arbitrary units.

To specifically test this hypothesis, we correlated a BLA response that encoded objectively different threat levels with trait anxiety scores. We found that a greater neural dissociation between cue-elicited BLA responses (high vs. low probability of upcoming shock, peak voxel activity) was significantly associated with lower trait anxiety (*r* = −.322, *p* = .045) (Figure 5B). Notably, this relationship remained significant when controlling for CMA activity (*r* = −.383, *p* = .018). However, there was no such relationship for CMA activity alone (*r* = .112, *p* = .492). This finding supports the notion that high trait-anxious individuals are characterized by a reduced discriminatory response to different threat levels in the BLA.

An impaired threat modulation of BLA activity could arise for two reasons. Anxious individuals might fail to activate BLA in response to highly threatening stimuli, or they might display elevated BLA responses to any threatening stimulus, regardless of its objective threat level. To arbitrate between these explanations, we correlated a BLA response to aversive cues regardless of threat level (collapsed across high- and low-probability trials, peak voxel activity) with individual trait anxiety scores. We found a significant positive correlation, i.e., a greater overall cue-elicited BLA response was associated with greater trait anxiety (*r* = .328, *p* = .041) (Figure 5A). Indeed, there was a positive relationship when testing for low and high threat cues separately (Supplemental Figure S8). This is in keeping with the idea that highly anxious individuals show heightened BLA activity for aversive cues regardless of their objective predictability. This suggests trait anxiety is associated with an elevated and undifferentiated threat response in BLA.

### Gender Differences

The human amygdala is thought to be a sexually dimorphic area (46), and anxiety disorders are more prevalent in women than men (47). Thus, we assessed potential gender differences across our sample of female (*n* = 22) and male (*n* = 17) participants.

Importantly, our sample was matched with regard to age (female: mean 24.5 ± 5.3, male: mean 25.6 ± 5.4; *p* = .532) and trait anxiety (female: mean 35.9 ± 7.1, male: mean 34.6 ± 10.7; *p* = .647). However, additional analyses (Supplemental Figure S9) showed that the discrimination for high and low levels of threat in the BLA was significantly stronger in female participants compared with male participants (*t*_38_ = 2.696, *p* = .010). Strikingly, this neural dissociation between threat levels showed a highly significant relationship to both subjective anxiety reports during the task (*r* = .552, *p* = .008) and trait anxiety (*r* = −.503, *p* = .017) in female participants, but not in male participants (*r* = −.082, *p* = .753 and *r* = −.290, *p* = .259, respectively). We found no such gender differences for outcome processing or any other comparison of our main results. This suggests that BLA responsivity to varying levels of threat was particularly pronounced in female participants, where greater dissociation between high and low levels of threat in BLA was related to greater threat-related dissociation of cues in subjective ratings and lower levels of trait anxiety.

## Discussion

We show that amygdala subregions, BLA and CMA, are distinctly engaged in processing of aversive experience. Specifically, the BLA encodes aversive expectations, where a dissociation across threat levels is mirrored by reported subjective anxiety. Importantly, BLA activity relates to trait anxiety, with more anxious subjects showing elevated and undifferentiated responses to threat, an effect particularly pronounced in female participants. Conversely, the CMA responds to aversive outcomes but is insensitive to aversive cues or their associated expectations.

In many human neuroimaging studies, participants are confronted with cues that vary not only in predictability (probability of shock) but also in uncertainty (absolute deviation from probability of shock equal to 50%) about upcoming aversive events. For example, previous studies often compared a partially reinforced aversive schedule with stimuli predicting complete safety [e.g., 48, 49, 50, 51]. This type of design renders it difficult to disentangle effects of predictiveness and uncertainty. Our task allowed us to control for uncertainty and in doing so shows that amygdala subregions play distinct roles in response to predictive stimuli. Most striking here is the observation that BLA activity scales with increasing levels of threat.

The BLA is anatomically well placed for processing environmental information about potential threat, as it receives dense connections from the thalamus and sensory association cortices 37, 52, 53, 54. Our findings complement previous accounts of the role of the BLA in learning about threat, demonstrating the BLA is important in detecting variable threat and predicting the occurrence of negative outcomes 15, 19, 20.

Activation in the amygdala to shock was primarily signaled in the CMA, highlighting its role in processing acutely imminent threat and pain 22, 23, 24. However, in contrast to the CMA, responses to aversive outcomes in the BLA were modulated by expectation, with enhanced activation for less predicted aversive events. This accords with prior neuroimaging studies showing unconditioned response diminution, i.e., reduced responses for expected versus unexpected aversive unconditioned stimuli, in the human amygdala 55, 56. Importantly, the BLA also displayed a greater attenuation in responsiveness for less predicted shock omission. Thus, responses at outcome to both aversive events and omission of such events in the BLA have the characteristics of a signed aversive prediction error (39). Such aversive prediction errors are known to play a crucial role in learning from aversive reinforcers such as pain 15, 57, 58. This finding extends previous studies that have shown the expression of amygdala prediction errors 27, 59 by demonstrating an anatomical specificity to this effect, an observation that is in accord with a similar finding in rodents 60, 61.

Consistent with prior evidence that the amygdala supports interoceptive emotional awareness 21, 62, 63, we found distinct relationships of the BLA and CMA with subjective experience. A greater neural dissociation within the BLA for threat levels was linked to a threat-related dissociation in reported subjective anxiety across individuals. Intriguingly, fluctuations in the CMA activity at the time of reporting were linked to subjective experience about previous anxiety states on a trial-by-trial basis. This indicates that retrospective reports about past aversive states are subject to an influence from current representation of outcomes in the CMA. This finding aligns with the role of the CMA as the major output center of the amygdala in generating behavioral responses to acute stressors 22, 23, 24.

An elevated BLA response to aversive cues in highly anxious individuals is consistent with prior neuroimaging findings that suggest a relationship between anxiety and amygdala hyperactivity 64, 65, 66, 67, 68. Importantly, anxious subjects showed a lack of discrimination for variable threat levels in the BLA, despite aversive cues being highly predictive. Interestingly, additional analyses showed that the association between greater trait anxiety and blunted threat discrimination in the BLA was particularly pronounced in female subjects. This finding demonstrates that high trait-anxious individuals display a failure to regulate BLA activity adequately in response to objectively different threat levels, supporting the notion that anxiety is associated with elevated and undifferentiated amygdala activity, potentially owing to a failure to adequately modulate its responses to objective features of the environment 43, 44, 45.

This link between a lack of discrimination of BLA responses and trait anxiety also concurs with previous work suggesting that high trait-anxious individuals do not accurately adjust expectations to reflect changes in environmental contingencies during aversive learning 69, 70. Such a failure to regulate BLA responses might in turn lead to an internal state of uncertainty about threat despite objectively predictable conditions and to increases in anxiety symptoms 45, 71. Overall, our findings complement previous studies indicating aberrant threat processing in amygdala putatively playing a role in the onset or maintenance of anxiety-related disorders 67, 68, 72.

### Limitations

First, our study provides multiple layers of evidence for the involvement of the BLA, but not the CMA, in responding to threat. However, in contrast to a strong dissociation between subregions for outcome processing, the comparison between subregions for aversive cues showed only a statistical trend. Thus, involvement of the CMA in processing of varying levels of threat cannot be fully ruled out. A second limitation of our study is the limited size and scope of the present sample. Although the observed relationships between amygdala activity and trait anxiety are consistent with prior work 43, 44, 45, future studies are needed to assess the reproducibility of these discoveries in larger samples 73, 74. Likewise, it will be fruitful to examine whether these relationships extend to individuals with more extreme levels of trait anxiety and to patients meeting diagnostic criteria for anxiety disorder (2).

### Conclusions

We show a functional dissociation within the human amygdala in relation to aversive processing. The CMA responds to aversive outcomes, whereas the BLA represents aversive events and expectations about those events. Moreover, BLA activity scales with increasing levels of threat, with more anxious individuals showing poorer discrimination across distinct threat levels. Our findings provide insight into how human amygdala subregions contribute to subjective anxiety, where an encoding of threat within the BLA emerges as a potential neurobiological mediator of vulnerability to anxiety disorders.
