# Supplementary material for: Distinct Processing of Aversive Experience in Amygdala Subregions
Source: Biol Psychiatry Cogn Neurosci Neuroimaging. 2020 Mar;5(3):291–300. doi: 10.1016/j.bpsc.2019.07.008 (PMC7059109; doi:10.1016/j.bpsc.2019.07.008)
Supplement: Supplemental Figures S1–S9 and Supplemental Tables S1–S3 [file mmc1.pdf]

Distinct Processing of Aversive Experience in Amygdala Subregions

Supplementary Information

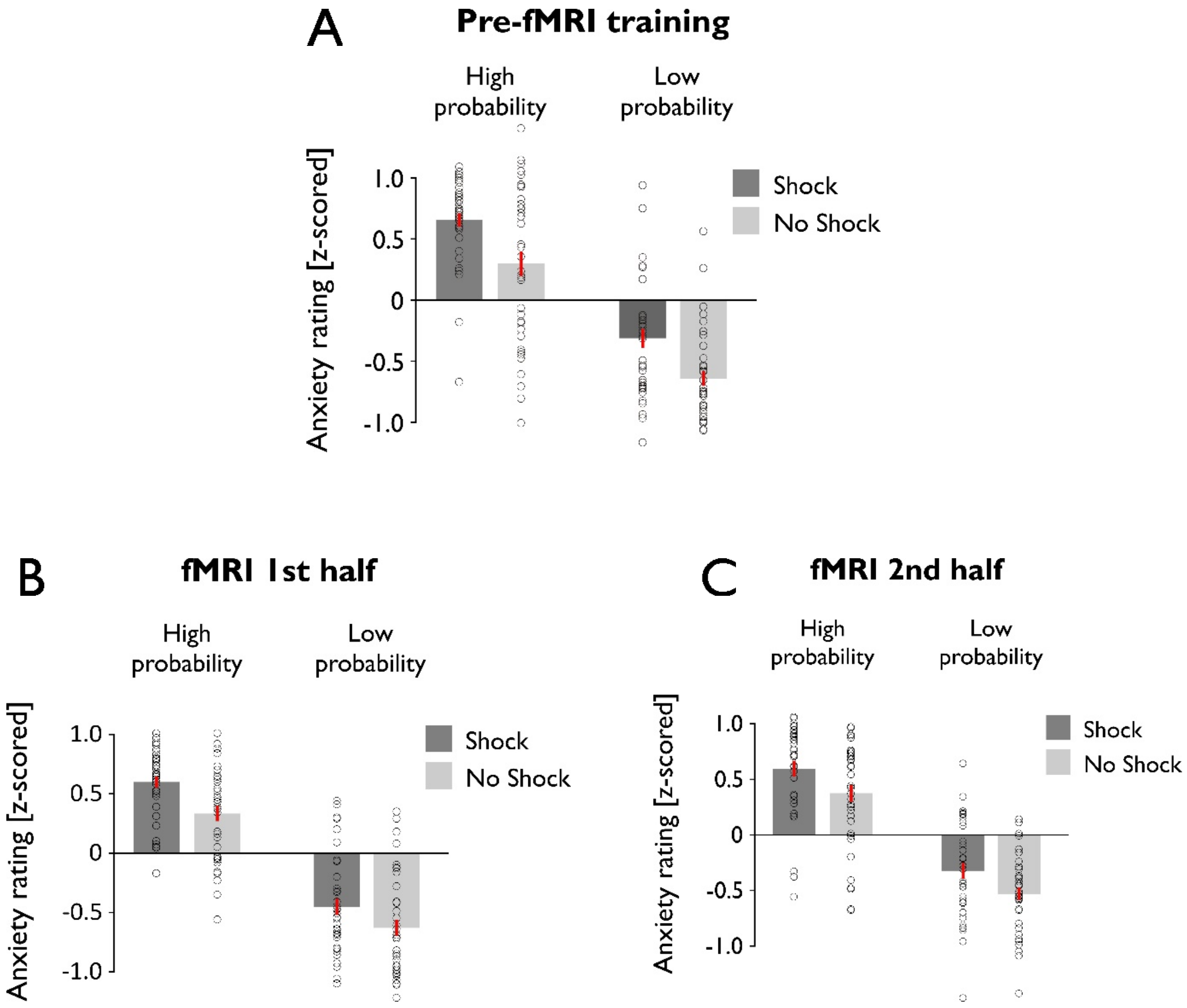

Supplementary Figure S1. *Anxiety ratings for pre-fMRI training and 1st and 2nd half of the fMRI experiment.* Subjective reports of remembered anticipatory anxiety at cue presentation. Anxiety ratings were influenced by both objective threat level in the cue period, i.e., greater for high vs. low expectation of upcoming shock, and were also biased by experienced outcomes, i.e., greater for shock vs. no shock trials. Error bars indicate SEM.

(A) Subjects had already learned to dissociate between two threat levels during training, as indicated by strong main effect of 'expectation' (high vs. low probability) [$F(1,38)=74.730$, $p<0.001$]. Similar to the main experiment, we also found a significant main effect of 'outcome' (shock vs. no shock) [$F(1,38)=23.621$, $p<0.001$], with no interaction [$F(1,38)=0.064$, $p=0.802$].

(B) & (C) Similar results were found when splitting the fMRI experiment in two separate halves:

1st half: 'expectation' (high vs. low probability): [$F(1,38)=109.074$, $p<0.001$], 'outcome' (shock vs. no shock): [$F(1,38)=23.828$, $p<0.001$], interaction: [$F(1,38)=2.499$, $p=0.122$].

2nd half: 'expectation' (high vs. low probability): [$F(1,38)=73.052$, $p<0.001$], 'outcome' (shock vs. no shock): [$F(1,38)=15.149$, $p<0.001$], interaction: [$F(1,38)=0.311$, $p=0.588$].

	**Entire Amygdala**			**BLA**					**CMA**						**BLA vs CMA**
	**voxel**			**voxel**			**mask**		**voxel**			**mask**			**mask**
	**[x y z]**	**T=**	**P_{FWE}=**	**[x y z]**	**T=**	**P_{FWE}=**	**beta=**	**P=**	**[x y z]**	**T=**	**P=**	**beta=**	**P=**		**P=**
**Cue**															
Expectation															
6mm	29 2 -23	4.17	0.028	29 3 -24	4.47	0.018	0.986	0.017	-	-	-	0.416	0.357		0.074
4.5mm	29 2 -21	4.30	0.024	29 3 -24	4.38	0.027	1.000	0.019	-	-	-	0.384	0.426		0.088
3mm	27 2 -20	4.54	0.019	27 2 -21	4.09	0.080	0.939	0.029	-	-	-	0.370	0.456		0.156
**Outcome**															
Shock															
6mm	26 -9 -12	8.28	<0.001	26 3 -21	5.83	<0.001	3.441	0.003	26 -9 -12	7.07	<0.001	8.662	<0.001		<0.001
	-20 -6 -12	7.20	<0.001	-24 -2 -20	4.79	0.007			-20 -6 -12	7.20	<0.001				
4.5mm	26 -9 -12	8.38	<0.001	26 3 -20	5.32	0.002	3.044	0.009	26 -8 -12	6.87	<0.001	9.222	<0.001		<0.001
	-24 -8 -12	6.80	<0.001	-24 -2 -20	4.69	0.011			-24 -8 -12	6.80	<0.001				
3mm	26 -9 -12	7.80	<0.001	24 3 -18	4.82	0.012	2.902	0.011	26 -6 -12	5.98	<0.001	9.790	<0.001		<0.001
	-26 -8 -12	7.15	<0.001	-24 -2 -20	4.52	0.026			-26 -9 -12	6.33	<0.001				
Expectation															
6mm	-27 -2 -26	4.41	0.016	-26 -2 -30	4.44	0.020	2.402	0.003	-	-	-	-0.138	0.886		0.003
4.5mm	-26 -3 -29	3.78	0.086	-26 0 -32	4.06	0.060	2.475	0.002	-	-	-	-0.276	0.796		0.005
3mm	30 -2 -24	3.86	0.102	-27 -5 -26	4.09	0.081	2.593	0.001	-	-	-	-0.335	0.779		0.009
**VAS**															
Self-reports															
6mm	-23 -6 -12	4.14	0.032	-	-	-	0.918	0.611	-23 -6 -12	4.14	0.011	4.125	0.032		0.014
4.5mm	-24 -5 -12	4.03	0.050	-	-	-	0.854	0.642	-24 -5 -14	3.94	0.021	4.237	0.047		0.021
3mm	-24 -5 -12	3.95	0.088	-	-	-	0.821	0.660	-24 -5 -14	3.86	0.034	4.223	0.078		0.045

Supplementary Table S1. *Main results for different smoothing kernels*
The main results reported in the manuscript (6mm smoothing kernel), as assessed with 4.5mm and 3mm smoothing kernels, respectively. Results are shown at the voxel-level (x, y, z coordinates in MNI-space), FWE-corrected for the respective bilateral mask, and for mean activation across the bilateral masks.
Cue Expectation: Activity at time of cue presentation for high vs. low probability of upcoming shock., Outcome Shock: Activity at time of outcome presentation for shock vs. no shock., Outcome Expectation: Activity at time of outcome presentation for low vs. high probability of shock., VAS Self-reports: Positive correlation between trial-by-trial activity at time of reporting and retrospective reports of subjective anxiety at cue presentation.
Entire amygdala = Independent bilateral amygdala mask from WFU PickAtlas toolbox, defined using the Automated Anatomical Labelling (AAL), CMA = Bilateral centromedial amygdala mask, BLA = Bilateral basolateral amygdala mask.

	Entire Amygdala					BLA				CMA			
	**[x y z]**	**T=**	**P_{FWE}=**	**% BLA**	**% CMA**	**[x y z]**	**T=**	**P_{FWE}=**	**% BLA**	**[x y z]**	**T=**	**P_{FWE}=**	**% CMA**
**Cue**													
Expectation	29 2 -23	4.17	0.028	37 [21-58]	0	29 3 -24	4.47	0.018	37 [18-57]	-	-	-	-
**Outcome**													
Shock	26 -9 -12	8.28	<0.001	0	19 [5-33]	26 3 -21	5.83	<0.001	40 [30-51]	26 -9 -12	7.07	<0.001	19 [5-33]
	-20 -6 -12	7.20	<0.001	0	36 [14-42]	-24 -2 -20	4.79	0.007	33 [23-55]	-20 -6 -12	7.20	<0.001	36 [14-42]
Expectation	-27 -2 -26	4.41	0.016	91 [58-94]	0	-26 -2 -30	4.44	0.020	85 [64-90]	-	-	-	-
**VAS**													
Self-reports	-23 -6 -12	4.14	0.032	0	31 [12-39]	-	-	-	-	-23 -6 -12	4.14	0.011	31 [12-39]

Supplementary Table S2. *Peak voxel statistics and cytoarchitectonic probabilities*
Results are shown at the voxel-level (x, y, z coordinates in MNI-space), p-values represent FWE-corrected statistics for the respective bilateral mask. Probabilities are computed from maximum probability maps, i.e., summary maps of different probabilistic cytoarchitectonic maps as implemented in the SPM anatomy toolbox.
Cue Expectation: Activity at time of cue presentation for high vs. low probability of upcoming shock., Outcome Shock: Activity at time of outcome presentation for shock vs. no shock., Outcome Expectation: Activity at time of outcome presentation for low vs. high probability of shock., VAS Self-reports: Positive correlation between trial-by-trial activity at time of reporting and retrospective reports of subjective anxiety at cue presentation.
Entire amygdala = Independent bilateral amygdala mask from WFU PickAtlas toolbox, defined using the Automated Anatomical Labelling (AAL), CMA = Bilateral centromedial amygdala mask, BLA = Bilateral basolateral amygdala mask.

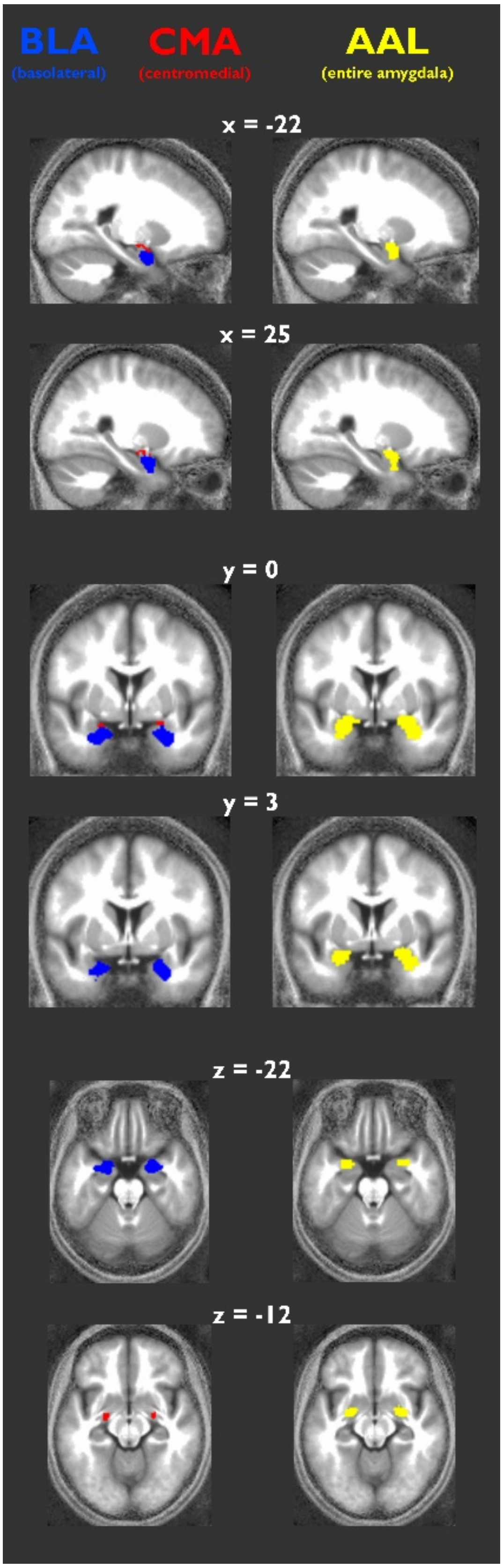

Supplementary Figure S2. *Visualisation of the ROIs superimposed on the mean normalized structural image.* CMA: Centromedial amygdala & BLA: Basolateral amygdala as derived from the SPM anatomy toolbox. AAL: Independent entire amygdala mask from WFU PickAtlas toolbox, defined using the Automated Anatomical Labelling (AAL) atlas. x, y, z coordinates in MNI-space.

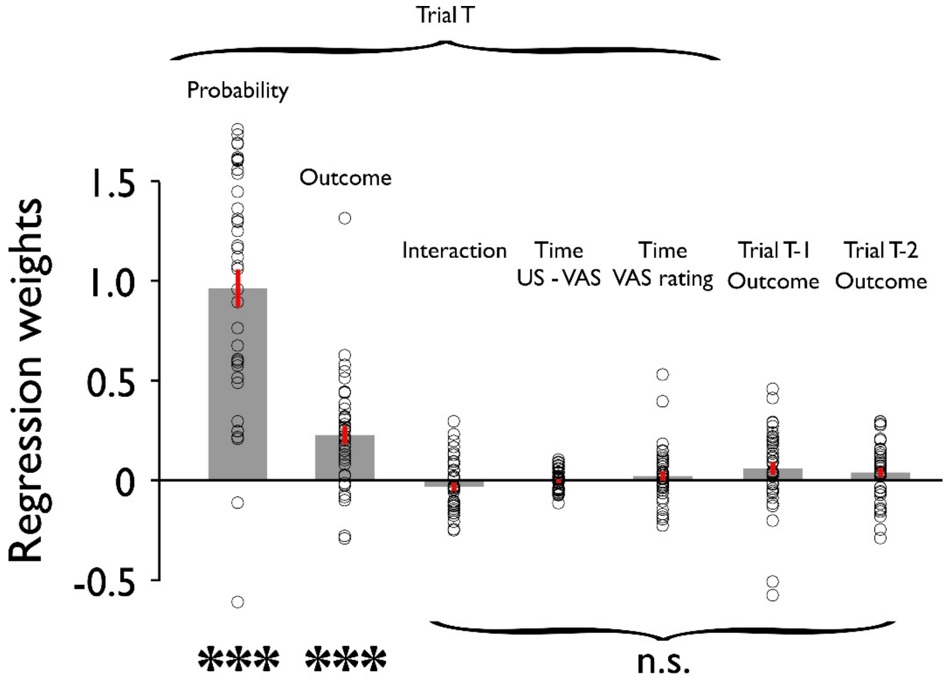

Supplementary Figure S3. *Trial-by-trial regression model of subjective anxiety ratings.*
To predict anxiety ratings on current trial T, we used (i) probability (high vs. low), (ii) outcome type (shock vs. no shock), and (iii) interaction term of the current trial T, whilst also testing for influence of (iv) elapsed time since outcome receipt (Time US-VAS), (v) time taken to report (Time VAS rating) and outcome type of previous trials, i.e., (vi) Trial T-1 and (vii) T-2.
Results confirmed the average effects reported in the manuscript:
Effect of probability (i): $p<0.001$; Effect of outcome (ii): $p<0.001$: Interaction n.s.: $p=0.131$, whilst additionally showing no effects of (iv): $p=0.720$, (v): $p=0.331$; (vi): $p=0.074$; (vii): $p=0.091$.

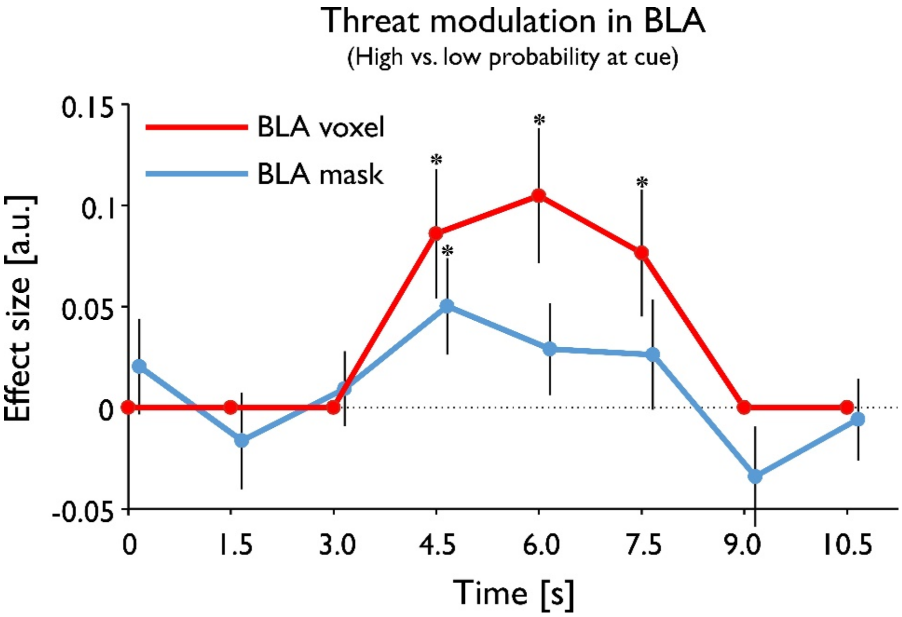

Supplementary Figure S4. *Finite impulse response analysis.*
BOLD response to aversive cues was modelled with a finite impulse response set consisting of a number of successive post-stimulus time bins ("mini-boxcars", 1.5s). Here, time represents post-stimulus onset time in seconds. This analysis revealed that BOLD response to threat, i.e., high vs. low probability at cue, showed a response pattern remarkably similar to a canonical HRF, with peak activity around 5-6s post-stimulus onset (as predicted by a canonical HRF). Note that this was true for both BLA peak voxel (informed from our conventional analysis, $p<0.05$) and mean activity averaged across the entire bilateral BLA mask.

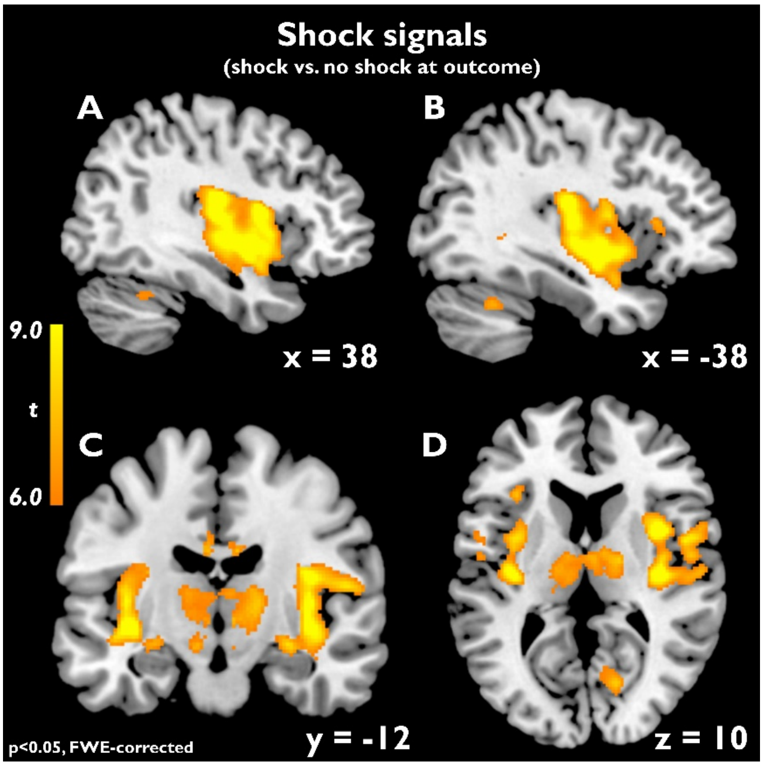

Supplementary Figure S5. *Whole-brain shock signals*
Contrast shock vs. no shock at outcome, $P_{FWE}<0.05$, whole-brain, corrected at voxel level.
x, y, z coordinates in MNI-space.

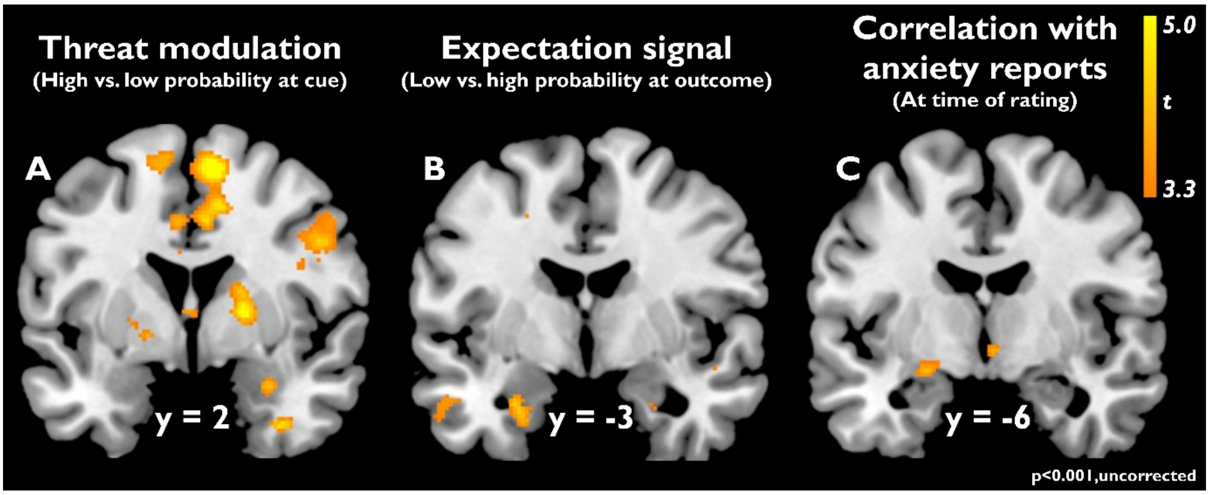

Supplementary Figure S6. *Whole-brain results at uncorrected height threshold, p<0.001.*
(A) Contrast high vs. low probability at cue.
(B) Contrast low vs. high probability at outcome.
(C) Positive correlation between trial-by-trial variability in activity at time of reporting on a visual analogue scale and retrospective reports of subjective anxiety at cue presentation.
Note that these slices correspond to the respective masked amygdala results presented in the main manuscript.
x, y, z coordinates in MNI-space.

**Region**	**[x y z]**	**T=**	**P_{FWE}=**
Primary Sensory Cortex (SI) – Right	38 -15 18	12.72	<0.001
Insula – Left	-36 -18 17	12.46	<0.001
Insula – Right	38 -2 -8	11.64	<0.001
Secondary Sensory Cortex (SII) – Right	56 -17 14	10.43	<0.001
Secondary Sensory Cortex (SII) – Left	-59 -26 24	9.11	<0.001
Precuneus – Left	-6 -69 33	8.94	<0.001
Periaqueductal Gray (PAG) – Left	-8 -27 -8	8.83	<0.001
Periaqueductal Gray (PAG) – Right	6 -30 -15	8.45	<0.001
Primary Sensory Cortex (SI) – Left	-47 -21 23	8.42	<0.001
Posterior Cingulate Cortex (PCC) – Left	-6 -21 30	8.32	<0.001
Thalamus – Right	8 -5 8	8.31	<0.001
Inferior Frontal Gyrus (IFG) – Left	-27 35 -14	7.83	0.001
Visual Cortex – Right	15 -72 11	7.83	0.001
Cerebellum – Right	6 -56 -35	7.73	0.001
Insula – Right	-35 23 12	7.64	0.002
Cerebellum – Right	2 -74 -15	7.62	0.002
Thalamus – Left	-14 -17 5	7.38	0.004
Middle Cingulate Cortex (MCC) – Right	5 18 35	7.28	0.006
Posterior Cingulate Cortex (PCC) – Left	-5 -41 41	7.25	0.006
Cerebellum – Left	-39 -54 -30	7.22	0.007
Centromedial Amygdala (CMA) – Left	-20 -6 -12	7.20	0.007
Visual Cortex – Left	-3 -83 6	7.17	0.008
Centromedial Amygdala (CMA) – Right	26 -9 -12	7.07	0.012
Anterior Cingulate Cortex (ACC) – Left	-5 18 30	7.05	0.012
Inferior Frontal Gyrus (IFG) – Right	33 32 5	6.53	0.048

Supplementary Table S3. *Whole-brain shock signals (corresponding to Supplementary Fig. 4)*
Local maxima derived from the contrast shock vs. no shock at outcome.
P_{FWE}<0.05, whole-brain, corrected at voxel level.

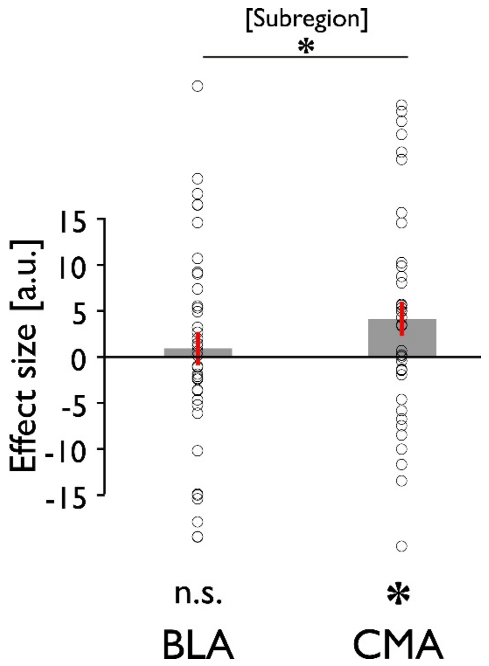

Supplementary Figure S7. *Relationship between activity at time of reporting and subjective anxiety.*
Mean betas for bilateral BLA and CMA masks for positive correlation between trial-by-trial-variability in activity and retrospective anxiety reports. * p<0.05, n.s. = not significant, a.u. = arbitrary units, error bars indicate SEM.

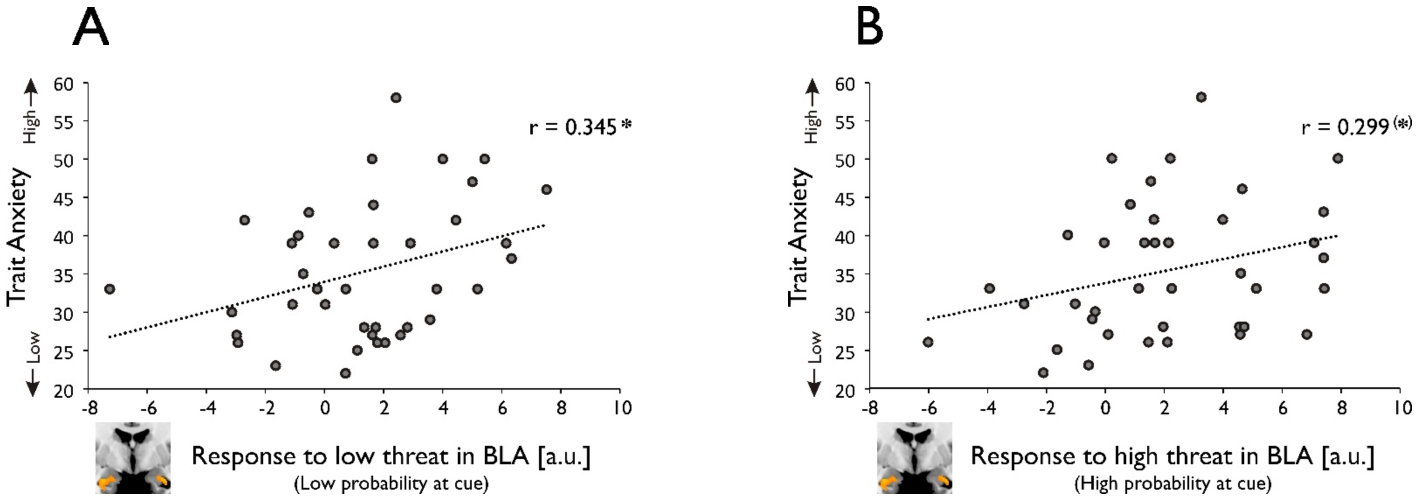

Supplementary Figure S8. *Threat signals in BLA and trait anxiety*
A greater cue-related BLA response to low (A) and (B) high levels of threat was associated with greater trait anxiety.
* p<0.05, (*) p=0.064; a.u. = arbitrary units.

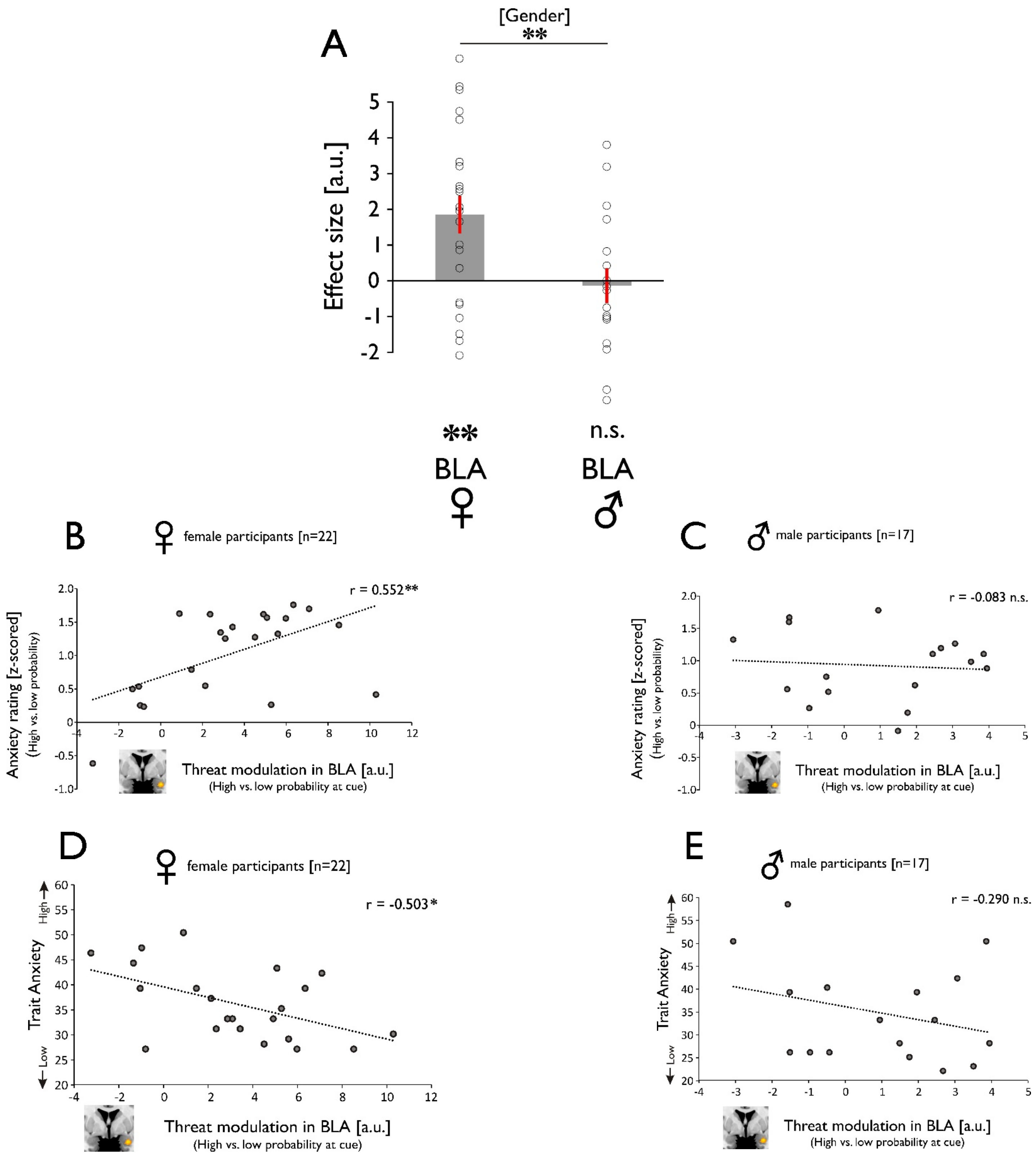

Supplementary Figure S9. *Sex differences in BLA threat modulation.*
(A) BLA activity at time of cue presentation scaling with enhanced objective threat levels, i.e., high vs. low probability of upcoming shock was significantly greater in female than male participants.
A greater neural difference between cue-elicited BLA responses (high vs. low probability of upcoming shock) was linked to a greater dissociation between threat levels in anxiety ratings (high vs. low probability of shock) in female (|B) but not in male participants (C). A greater neural difference between cue-related BLA responses (high vs. low probability of upcoming shock) was associated with lower trait anxiety in female (|D) but not in male participants (E). ** $p<0.01$, * $p<0.05$, n.s. = not significant, a.u. = arbitrary units, error bars indicate SEM.
